# Predicting neurological deficit in patients with spinal tuberculosis – A single-center retrospective case-control study

**DOI:** 10.1051/sicotj/2021002

**Published:** 2021-03-05

**Authors:** Samarth Mittal, Gagandeep Yadav, Kaustubh Ahuja, Syed Ifthekar, Bhaskar Sarkar, Pankaj Kandwal

**Affiliations:** 1 Senior Resident, AIIMS Rishikesh 249203 Rishikesh Uttarakhand India; 2 Department of Orthopaedics, AIIMS Rishikesh 249203 Rishikesh Uttarakhand India; 3 Assistant Professor, AIIMS Rishikesh 249203 Rishikesh Uttarakhand India; 4 Additional Professor, Consultant Spine Surgeon, AIIMS Rishikesh 249203 Rishikesh Uttarakhand India

**Keywords:** Spinal tuberculosis, Pott’s paraplegia, Neurologic deficit, Risk factors

## Abstract

*Background*: Identifying the risk factors for the neurological deficit in spine tuberculosis would help surgeons in deciding on early surgery, thus reducing the morbidity related to neurological deficit. The main objective of our study was to predict the risk of neurological deficit in patients with spinal tuberculosis (TB). *Methods*: The demographic, clinical, radiological (X-ray and MRI) data of 105 patients with active spine TB were retrospectively analyzed. Patients were divided into two groups – with a neurological deficit (*n* = 52) as Group A and those without deficit (*n* = 53) as Group B. Univariate and multivariate logistic regression analysis was used to predict the risk factors for the neurological deficit. *Results*: The mean age of the patients was 38.1 years. The most common location of disease was dorsal region (35.2%). Paradiscal (77%) was the most common type of involvement. A statistically significant difference (*p* < 0.05) was noted in the location of disease, presence of cord compression, kyphosis, cord oedema, loss of CSF anterior to the cord, and degree of canal compromise or canal encroachment between two groups. Multivariate analysis revealed kyphosis > 30° (OR – 3.92, CI – 1.21–12.7, *p* – 0.023), canal encroachment > 50% (OR – 7.34, CI – 2.32–23.17, *p* – 0.001), and cord oedema (OR – 11.93, CI – 1.24–114.05, *p* – 0.03) as independent risk factors for predicting the risk of neurological deficit. *Conclusion*: Kyphosis > 30°, cord oedema, and canal encroachment (>50%) significantly predicted neurological deficit in patients with spine TB. Early surgery should be considered with all these risk factors to prevent a neurological deficit.

## Introduction

Tuberculosis (TB) of the spine is most common among osteoarticular tuberculosis, accounting for about 50% of cases [1]. The WHO has reported an estimated incidence of around 10 million (range, 9.0–11.1 million) new cases in its global tuberculosis report in 2019 [2]. Delay in diagnosis or management of spondylodiscitis may cause serious long-term morbidity [3]. Spinal TB has a slow and insidious onset, presents with a constellation of symptoms like back pain, malaise, night sweats, fever, and/or weight loss. Neurological deficit is one of its most feared complications, can occur in 10–30% of cases [4–7]. It can present as; (a) paraplegia of active disease (early onset) and (b) paraplegia of healed disease (late-onset) [6, 7]. Usual causes of neurological complications in caries spine include mechanical compression by an abscess, tubercular sequestra, granulation tissue, caseous material, localized pressure by internal gibbus, and/or pathological subluxation/dislocation of the vertebrae. The spinal cord can undergo inflammatory edema and intrinsic changes like cord atrophy, gliosis, syrinx, or myelomalacia all accounting for neural deficit [4]. More than one factor may be responsible at a time. Also, the location of the disease bears importance concerning susceptibility to neural deficit [4]. The insidious course of the disease, nonspecific inflammatory markers, lag of radiological findings on X-ray by 3–4 months can lead to progression of the disease, causing the neurological deficit. The recovery pattern after neurological deficit has developed in cases of spinal TB is unpredictable.

Some authors have correlated CT and MRI findings to neurological deficit [8, 9] or clinical course of disease [10, 11] in Pott’s spine. Diffusion tensor imaging (DTI) has also been investigated to show correlation with a neural deficit in TB spine in past, however epidural collection and its organized inflammatory tissue precludes accurate assessment of diffusion characteristics of the compressed cord [12]. Very few studies in the literature have attempted to predict risk factors for the neurological deficit in the TB spine [13–15]. Sparse literature and lack of objective measurements of canal compression/encroachment in MRI in previous studies made us to conduct this study to identify the risk factors for the neurological deficit which would help surgeons in making decisions for early surgery and thus avoiding the various complications/morbidity arising due to neurological deficit in cases of spinal TB.

## Material and methods

A retrospective case-control study was conducted in the department of orthopaedics after institutional ethical committee approval. Clinical and radiological data of 105 patients with spinal tuberculosis confirmed on basis of histopathology and/or culture who presented to our institution from 2016–2019 was analyzed. Patients with implants not compatible with MRI, active malignancy, healed disease, paraplegia of late-onset, spinal tumour syndrome were excluded from our study. Neurological deficit was graded as per the ASIA Impairment scale [16]. Patients with a neurological deficit (ASIA grade A, B, C, D) were included in Group A (*n* = 52) and those without deficit (neurologically intact [NI]) in Group B (*n* = 53). Data included patient demographic characteristics, ASIA grade, location of disease (cervical, thoracic, thoracolumbar, lumbar, lumbosacral), type of involvement (anterior subligamentous, central, paradiscal, posterior, panvertebral), number of vertebral bodies affected, presence or absence of skip lesion, posterior element involvement.

Anterior vertebral Height loss percentage (AVH loss) and Kyphotic angle were recorded from X-rays. AVH loss percentage was calculated by the method described by Jain et al. [17]. In this method, the mean of the anterior heights of the upper and lower normal vertebral bodies were taken as presumed anterior vertebral height (AVH). The loss of the anterior height of affected vertebrae was calculated by subtracting the measured height from the presumed height for each vertebra, which was then summed to obtain the total height loss. AVH loss percentage was then calculated as − Total height loss/Presumed AVH × 100. Kyphotic angle was calculated using cobbs method [18] as the angle between the upper border of upper normal vertebrae and lower border of lower normal vertebrae. Kyphosis more than 30° were compared between two groups as a predictive risk factor [15, 19].

**Figure 1:**
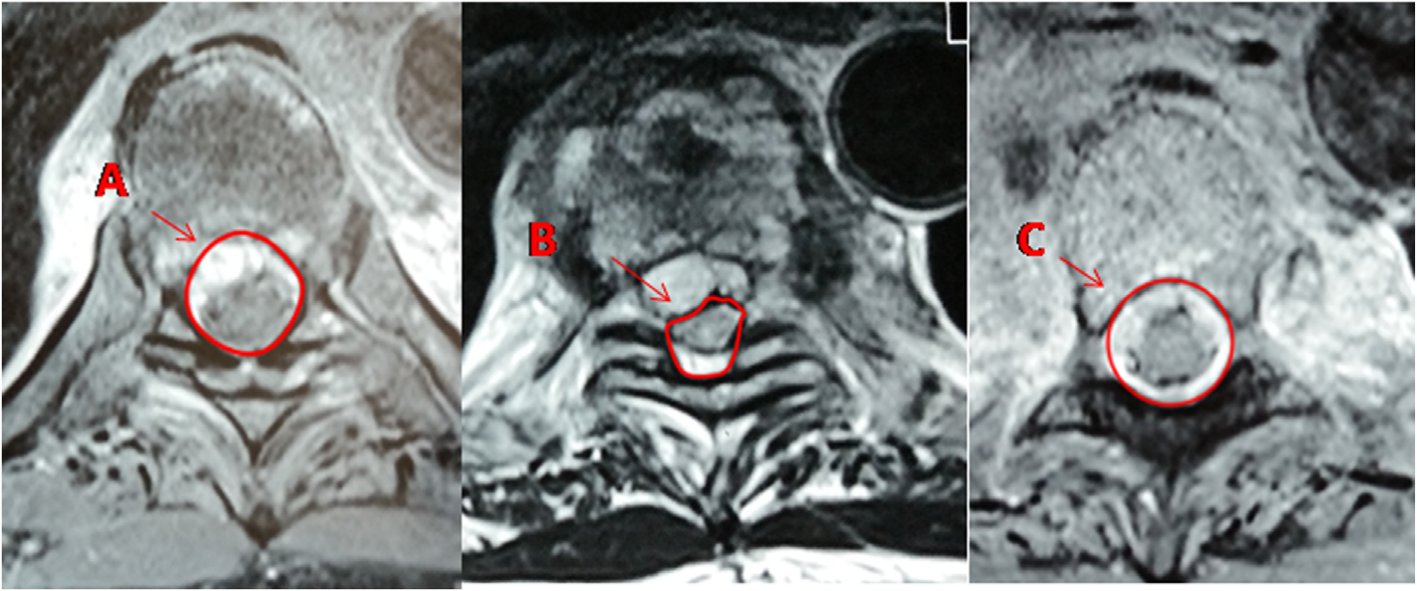
Measurement of canal encroachment in axial section of MRI. *A* = cross-sectional area of canal one level above the site of maximum compression, *B* = area occupied by spinal cord at maximum compression, *C* = cross-sectional area of canal one level below site of maximum compression. Average canal area (*D*) = *A* + *C*/2. Percentage of canal encroachment = *D* − *B*/*D* × 100.

MRI was reviewed by two independent observers to decrease interobserver variability and a common agreement was reached. Observations in MRI included the cause of compression (abscess, granulation tissue, disc, vertebral body bulge, or combination of above), Loss of CSF anterior and posterior to the cord, cord signal changes, and canal encroachment. Canal encroachment area was calculated on the axial image at the site of maximum compression using Surgimap software. Percentage of canal encroachment was calculated by the formula: The average of canal area − Spinal cord area/Average of canal area × 100. Average canal area was calculated by taking the average canal area of proximal and distal vertebrae to the diseased segment (Figure 1) [8].

Logistic regression analysis was used to predict risk factors for the neurological deficit. As the spinal cord ends at the level of L1 vertebrae, we excluded the cases with “below conus disease” (lumbar and lumbosacral spine cases) for predicting the effect of spinal cord changes (cord oedema, loss of CSF anterior and posterior to the cord) along with other risk factors on the neurological deficit while performing logistic regression analysis.

### Statistical analysis

All data were analyzed using IBM, SPSS statistics version 17.0. Descriptive statistics such as mean, standard deviation, percentages, frequencies, cross-tabulations were used for baseline characteristics. An independent *t*-test was used for comparing quantitative data while non-parametric tests were used for comparing categorical data. Significance was considered at a *P*-value < 0.05. Univariate and multivariate logistic regression analysis was performed. Potentially predictive variables found significant in univariate analysis (*p* < 0.2) [20] were included in multivariate analysis. Methods using forward stepwise logistic regression was performed to determine statistically significant risk factors (*p* < 0.05).

## Results

**Figure 2:**
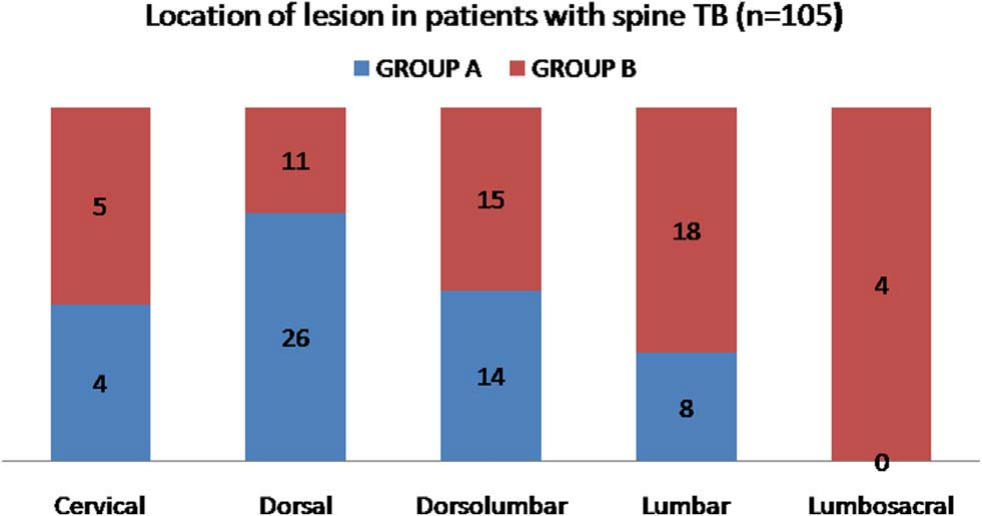
Location of lesion in patients with spine TB (*n* = 105). Group A – patients with neurological deficit (*n* = 52). Group B – patients without deficit (*n* = 53).

**Figure 3:**
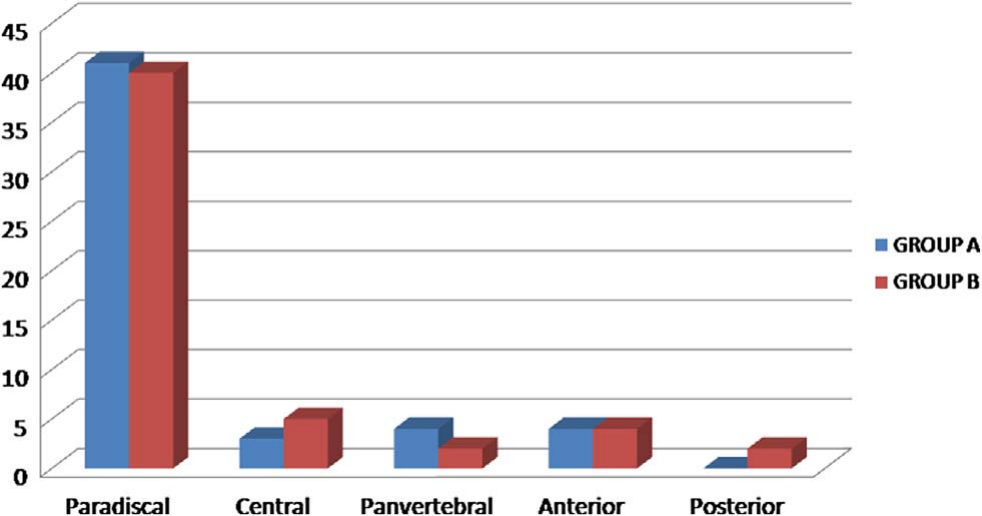
Type of involvement of lesion in patients with spinal tuberculosis (*n* = 105). Group A – patients with neurological deficit (*n* = 52). Group B – patients without deficit (*n* = 53)

Out of 105 active TB cases who were enrolled in our study, 40/105 (38.1%) were male while 65/105 (61.9%) patients were female. The mean age of the patients was 38.1 years (range, 12–69 years). The most common location of disease was the dorsal region (35.2%) followed by Dorsolumbar (27.6%), Lumbar (24.8%), Cervical (8.6%), and Lumbosacral (3.8%) (Figure 2). Paradiscal was the most common type of involvement seen in 77.1% cases (*n* = 81) followed by Central type (*n* = 8, 7.6%), Anterior subligamentous (*n* = 8, 7.6%), Pan vertebral (*n* = 6, 5.7%), and Posterior type (*n* = 2, 1.9%) (Figure 3). Posterior element involvement was seen in 8 cases (7.6%). Skip lesions were noted in only 5.7% (*n* = 6) cases. The average number of vertebral bodies involved was 2.39, ranging from 1 to 7 in number. The descriptive data of patients in the two groups is summarized in Table 1. A statistically significant difference (*P* < .05) was noted between two groups in the location of disease, presence of cord compression kyphotic deformity, cord oedema, loss of anterior CSF around the cord, and canal encroachment (Table 1).

**Table 1:** Descriptive data of the patients (*N* = 105).

Characteristic	Group A (*n* = 52)	Group B (*n* = 53)	*P*-value
Age (mean [SD])	40.31 (17.03)	35.56(15.77)	0.401
Sex (*n*, [%])			0.939
Male	20 (38.5%)	20 (37.7%)	
Female	32 (61.5%)	33 (62.3%)	
Location (*n*, [%])			**0.007**
Cervical	4 (7.7%)	5 (9.4%)	0.75
Dorsal	26 (50.0%)	11 (20.8%)	0.002
Dorsolumbar	14 (26.9%)	15 (28.3%)	0.874
Lumbar	8 (15.4%)	18 (34.0%)	0.027
Lumbosacral	0	4 (7.5%)	0.043
Neurological deficit (*n*, [%])			
Grade A	13 (25.0%)		
Grade B	5 (9.6%)		
Grade C	14 (26.9%)		
Grade D	20 (38.5%)		
Grade E		53 (100%)	
Type of involvement (*n*, [%])			0.53
Paradiscal	41 (78.8%)	40 (75.5%)	0.681
Central	3 (5.8%)	5 (9.4%)	0.479
Panvertebral	4 (7.7%)	2 (3.8%)	0.387
Anterior	4 (7.7%)	4 (7.5%)	0.978
Posterior	0	2 (3.8%)	0.157
Posterior element (*n*, [%])			
Involved	5 (9.6%)	4 (7.5%)	0.705
Compression (*n*, [%])			**0.001**
Abscess	44(84.6%)	35(66%)	
Granulation tissue	2 (3.8%)	1 (1.9%)	
Disc	3 (5.8%)	0	
Vertebral body bulge	2 (3.8%)	5 (9.4%)	
None	1 (1.9%)	12 (22.6%)	
X-ray (mean [SD])			
Number. of vertebra	2.46 (1.07)	2.32 (1.16)	0.523
AVH loss (%)	43.45 (18.90)	37.02 (21.22)	0.8
Kyphosis (°)	26.92 (12.67)	21.72 (11.89)	**0.032**
MRI (*n*, [%])			
Loss of anterior CSF	31/44 (70.5%)	7/31 (22.5%)	**<0.001**
Loss of posterior CSF	3/44 (6.8%)	1/31 (3.2%)	0.495
Cord oedema	12/44 (27.3%)	1/31 (3.2%)	**0.007**
Canal encroachment (%) (mean, [SD])	56.16 (18.13)	34.52 (23.42)	**<0.001**

Bold values represent *p*-values that are significant (*p* < 0.05).

**Figure 4:**
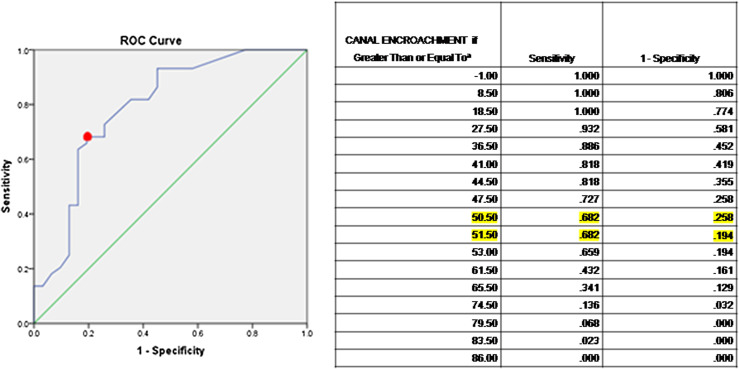
Receiver operating characteristic (ROC) curve to find the threshold value of canal encroachment above which it had the maximum association with neurological deficit. *Y*-axis (sensitivity) represents true positive while *X*-axis (1-specificity) represents false positives. The threshold value is represented by red dot.

Age, location of disease (dorsal), AVH loss, kyphosis > 30°, loss of CSF anterior to the cord, cord oedema, and canal encroachment were found to be possible risk factors for the neural deficit in univariate analysis (*p* < 0.2) in our study (Table 2). ROC Curve was plotted to assess the overall accuracy and the value of canal encroachment above which had a maximum association with a Neurological deficit. Canal encroachment value equal to or greater than 50.5–51.5% as the cutoff was best associated with neurological deficit (Figure 4). Multivariate logistic regression analysis showed kyphosis > 30° (OR – 3.92, CI – 1.21–12.7, *p* – 0.023), canal encroachment > 50% (OR – 7.34, CI – 2.32–23.17, *p* – 0.001), and cord oedema (OR – 11.93, CI – 1.24–114.05, *p* – 0.03) as independent risk factors for neurological deficit with Nagelkerke *R* ^2^ ranging from 0.26 to 0.42 (Table 3).

**Table 2:** Univariate analysis.

Possible risk factors	Crude odds ratio	95% confidence interval	*P*-value
Age	1.03	1.00–1.06	0.07
Sex (%)	1.22	0.47–3.17	0.682
Location			0.14
Cervical			
Dorsal	2.95	0.66–13.13	0.155
Dorsolumbar	1.17	0.26–5.24	0.841
Type of involvement			0.37
Anterior			
Central	0.53	0.06–4.9	0.58
Panvertebral	2.7	0.27–25.63	0.39
Paradiscal	2.6	0.52–12.85	0.24
Posterior	0.001	0.00–inf	0.99
Posterior element	1.45	0.25–8.46	0.68
Compression	>1000	0.00–inf	0.99
X-ray			
No. of vertebrae	1.07	0.73–1.56	0.722
AVH loss	1.024	0.99–1.05	0.06
Kyphosis > 30°	2.68	1.009–7.1	**0.04**
MRI			
Loss of anterior CSF	6.86	2.44–19.26	**0.001**
Loss of posterior CSF	2.2	0.22–22.15	0.505
Cord oedema	11.25	1.38–91.86	**0.024**
Canal encroachment	1.06	1.03–1.09	**0.001**

Bold values represent *p*-values that are significant (*p* < 0.05).

**Table 3:** Multivariate analysis including showing Kyphosis > 30°, canal encroachment (>50%) and Cord oedema as independent risk factors associated with neurological deficit *B* – unstandardized beta, *SE* – standard error of regression, Wald- type of statistic used in logistic regression analysis, *df* – degrees of freedom, Sig. – significance value, Exp(*B*) – odds ratio, CI – confidence interval.

	*B*	*SE*	Wald	*df*	Sig.	Exp (*B*)	95% CI for Exp (*B*)	
							Lower	Upper
Step 1								
Canal encroachment > 50%	2.037	.532	14.657	1	.000	7.667	2.702	21.751
Constant	−.651	.356	3.338	1	.068	.522		
Step 2								
Canal Encroachment > 50%	1.919	.551	12.125	1	.000	6.817	2.314	20.083
Oedema	2.174	1.118	3.781	1	.052	8.797	.983	78.736
Constant	−.843	.380	4.929	1	.026	.430		
Step 3								
Canal encroachment > 50%	1.994	.586	11.554	1	.001	**7.342**	2.326	23.175
Oedema	2.480	1.152	4.635	1	.031	**11.935**	1.249	114.054
Kyphosis > 30°	1.367	.600	5.198	1	.023	**3.924**	1.211	12.711
Constant	−1.485	.508	8.529	1	.003	.227		

Bold values in Table 3 represent Odds ratio of variables that are significant.

## Discussion

TB spine is a medical disease with reserved surgical indications [4]. Problems arising from immobilization due to neurological deficit can complicate the disease and might become greater problems than the primary disease itself. Thus, it becomes important to identify the clinic-radiological risk factors beforehand that can predict the worsening of neurology so that timely surgical intervention could be added to the medical treatment. As per the Global tuberculosis report 2019, India accounts for 27% of the global total making it among the top eight countries which accounts for two-thirds of the global tuberculosis burden [2]. Tuberculosis of the spine is an important health problem, especially in developing countries like India due to poverty, undernutrition, and the immigrant population [21]. TB affects both sexes of all age groups. In our study, the mean age of the patients was 38.1 years. The dorsal spine was the most common region affected (35.2%) followed by Dorsolumbar (27.6%) region. Literature has also shown the dorsal [11, 22] or dorsolumbar region [23, 24] to be the most commonly affected site. Paradiscal type of involvement is the most common type of presentation reported [14, 21, 25], which was also found in our study (77.1%).

A large number of patients still present in advanced stages of the disease for the first time. Delay in the start of treatment leads to the progression of the natural course of the disease and worsening of neurological deficit in Spinal TB, ranges from 10 to 43% in various studies [25, 26]. In our study 49.5% of patients presented with a neurological deficit which is likely due to complicated cases referred for management in our tertiary care center. The cause of deficit in active TB cases can be mechanical compression by an abscess, sequestra, caseous tissues, debris, and granulation tissue. Mechanical instability, inflammatory oedema of the cord, intrinsic cord changes can also occur leading to neural deficit [6]. We considered all these risk factors while evaluating for the neurological deficit in our study. The limitation of our study is the small sample size in each group after the exclusion of lumbar and lumbosacral cases for multivariate analysis. Also, the ASIA grading has its own limitation that it does not account for pain, spasticity, or dysesthesia that might result from spinal cord injury or compression.

Patients with neurological deficit not showing improvement on 3–4 weeks of conservative treatment or getting to worsen in neural status should undergo surgical decompression [4]. Patients who present with rapid onset paraplegia, severe paraplegia require surgical decompression. Also, pre-treatment long segment (4 or more vertebral disease) disease with kyphosis or kyphotic deformity > 60° at presentation are candidates for deformity correction and decompression. Mechanical instability due to panvertebral disease requires instrumented stabilization and decompression. Diagnostic dilemmas in patients not showing response to antitubercular treatment can be considered for decompression and procurement of tissue for diagnosis [4].

Since MRI allows better visualization of neural structure, central canal, and foramen, we used axial images at the site of maximum compression to document the degree of cord compression measured as canal encroachment. In our study, we found that even canal encroachment of 73.5% in a case of dorsal Potts spine had intact neurology. This could be explained due to the plasticity of the cord and physiological reserve to withstand gradual compression. Jain et al reported that even 76% of canal encroachment in CT scan of active TB cases was compatible with intact neurology, with no other insult like vascular cause or instability exists. However, the deficit can occur with a lesser degree of encroachment if more than one factor is present [8].

**Figure 5:**
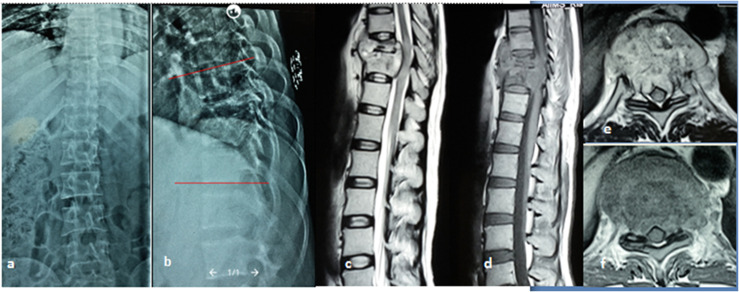
A 42-year-old female diagnosed as case of Potts spine D9–D10 with neurological deficit. (a) X-ray AP and (b) lateral view showing fuzziness of end plates of D9–D10 with kyphosis of 16° measured using cobs method. (c) MRI sagittal T2WI, (d) sagittal T1WI, (e) axial T2WI, (f) axial T1WI showing paradiscal involvement of D9–D10 vertebral bodies with endplate destruction and altered marrow signal intensity, prevertebral and epidural collection causing loss of anterior CSF around cord and cord compression.

In our study, we found that patients with neurological deficit had a statistically significant difference in location of disease, kyphosis, presence of cord compression, cord oedema, loss of CSF anterior to the cord, and canal encroachment or degree of canal compromise as compared to patients with NI (Figure 5). These findings were consistent with studies done previously. Subhadrabandhu et al. [27] reported the cephalad level of infection and vertebral body loss to be more commonly associated with neurological deficit. Sae-Jung et al. [15] found the location of disease, cord oedema, notable cobbs angle, and epidural abscess to be associated with neurological deficit. A significant difference was reported between the two groups in the location of vertebrae involved and spinal compression by Wang and Yang [13]. In our study, mean kyphosis, AVH loss, and canal encroachment were higher in the neurological deficit group. Cord oedema was observed in MRI of 13 patients, 12 of them had a complete neurological deficit (ASIA grade A) while one patient had intact neurology. Thoracic location of disease was more commonly associated with neurological deficit group (*p* – 0.002).

Multivariate logistic regression analysis in our study revealed that kyphosis > 30°, cord oedema, and canal encroachment, which also implies a degree of spinal canal compression, as independent risk factors for predicting neurological deficit in patients with spine TB. Compared to previous studies (Table 4), Wang et al. in a retrospective study of 329 patients with spine TB reported age (elderly), worsening of sickness, location (cervical or lumbar vertebrae), and spinal compression as predictive risk factors of Lower extremity motor or sensory deficit [13]. Age and location of disease came out to be predictors in univariate analysis in our study but they were not found as independent risk factors in multivariate analysis. Moreover, the dorsal location of the disease was more commonly associated with a deficit in our study as compared to cervical or lumbar vertebrae. Singh et al. [14] in their study on 40 TB spine patients with neural deficit found cord oedema and cord compression as predictive risk factors. Sae-Jung et al. [15] identified signal cord change (OR – 3.31) and notable cobbs angle (>30°) (OR – 2.62) as predictive factors for neurological deterioration in his study of 125 patients with spine TB.

**Table 4:** Comparison of studies in literature predicting neurological deficit in TB spine.

Authors	Year	Study design	No. of patients	Predictors of neurological deficit			
					Odds ratio	95% confidence interval	*P*-value
Wang and Yang [13]	2016	Retrospective study	329	Age	1.761	1.227–2.526	0.002
				Worsening of sickness	1.910	1.161–3.141	0.011
				Location (thoracic vs. cervical)	0.204	0.063–0.662	0.008
				Spinal compression	1.672	1.020–2.741	0.042
Singh et al. [14]	2019	Prospective study	40	Cord compression	14.67	1.09–197.96	0.043
				Cord oedema	21.42	1.68–272.72	0.018
Sae-Jung et al. [15]	2019	Retrospective study	125	Cobbs angle > 30	2.62	1.03–6.67	0.04
				Signal cord change	3.31	1.50–7.3	0.003
Our study	2020	Retrospective study	105	Kyphosis > 30°	3.92	1.21–12.7	0.023
				Canal encroachment > 50%	7.34	2.32–23.17	0.001
				Cord oedema	11.93	1.24–114.05	0.03

The clinical importance of findings observed in our study is that patients with spinal tuberculosis should undergo MRI of the spine at the earliest to look for the location of disease, vertebral body destruction, kyphosis, cord compression, CSF anterior to the cord, cord signal changes as all these findings are significantly associated with neurological deficit in patients with spine TB. Kyphosis > 30°, cord oedema, and canal encroachment > 50% are important predictors of neurological deficit as evident by our study, hence should always be accounted for while planning management for a spine tuberculosis case. Cord oedema had the highest odds for neurological deficit among three predictors. The odds for developing neurological deficit are 12 times higher in patients presenting with cord oedema. Patients with kyphosis > 30° have a 3.9 times risk of developing neural deficit than those who present with cobs angle < 30°. Risk estimates of developing neural deficit are 7.3 times higher in patients having canal compromise of more than 50%.

## Conclusion

Tuberculosis of the spine is a medical disease with reserved indications for surgery, but identifying risk factors beforehand can help doctors to take a decision for early surgery to prevent morbidity due to neurological deficit. Hence, planning the management of spine tuberculosis case warrants attention towards the presence of cord oedema, kyphosis more than 30°, and degree of canal compromise or canal encroachment as they are independent predictors of neurological deficit in case of tuberculosis of the spine.

## Declarations

### Funding

None.

### Avaliablity of data and material

The datasets generated and/or analysed during the current study are available from the corresponding author on reasonable request.

### Code availabilty

Not applicable.

### Authors contribution

1. Conceptualization: Dr. Pankaj kandwal.
2. Methodology: Dr. Pankaj Kandwal, Dr. Samarth Mittal, Dr. Bhaskar Sarkar.
3. Formal analysis: Dr. Samarth Mittal, Dr. Gagandeep, Dr. Kaustubh Ahuja, Dr. Syed Ifthekar.
4. Writing – original draft: Dr. Samarth Mittal, Dr. Gagandeep.
5. Writing – review and editing: Dr. Pankaj Kandwal, Dr. Samarth Mittal, Dr. Bhaskar Sarkar, Dr. Kaustabh Ahuja.

## Conflict of interest

All authors certify that they have no affiliations with or involvement in any organization or entity with any financial interest (such as honoraria; educational grants; participation in speakers’ bureaus; membership, employment, consultancies, stock ownership, or other equity interest; and expert testimony or patent-licensing arrangements), or non-financial interest (such as personal or professional relationships, affiliations, knowledge or beliefs) in the subject matter or materials discussed in this manuscript.
